# Preliminary Study of Differential circRNA Expression and Investigation of circRNA–miRNA–mRNA Competitive Endogenous Network in Rumen Acidosis of Holstein Cattle

**DOI:** 10.3390/ani15101472

**Published:** 2025-05-19

**Authors:** Saeid Neysi, Jamal Fayazi, Hedayatollah Roshanfekr, Ikhide G. Imumorin

**Affiliations:** 1 Department of Animal Science, Agricultural Sciences and Natural Resources, University of Khuzestan, Ahvaz 6341773637, Iran; saeid.neysi268@asnrukh.ac.ir (S.N.); roshanfekr_hd@asnrukh.ac.ir (H.R.); 2 California State University Biotechnology Program (CSUBIOTECH), College of Science, San Diego State University, San Diego, CA 92182-0001, USA

**Keywords:** RNA sequencing, miRNA, transcription factors, inflammation pathways, SARA

## Abstract

Rumen acidosis is a widespread digestive disorder in livestock, causing inflammation and lowering animal performance. Unraveling its molecular mechanisms is vital for improving cattle health and welfare. Circular RNAs (circRNAs) are noncoding RNAs functioning as miRNA or protein sponges. This study employed high-throughput RNA sequencing to identify differentially expressed (DE) circRNAs in subacute rumen acidosis (SARA) in Holstein cattle, revealing 65 DE-circRNAs. We constructed a competitive endogenous RNA (ceRNA) network comprising 57 circRNAs, 14 miRNAs, and 22 mRNAs. Key hub nodes included circRNAs (8:69996068-69996853, 16:2614111-2615445, 5:109525933-109531380, 20:63115665-63116774), miRNAs (bta-miR-146b, bta-miR-181a, bta-miR-223, bta-miR-130b), and mRNAs (SLC2A3, SOCS3, DLC1, ARRDC4). Examination of hub circRNA host genes identified 30 DE transcription factors (TFs). Functional and pathway enrichment analysis pinpointed inflammation and immune response pathways, such as NF-kappa B and TNF signaling. This pioneering study offers the first circRNA expression profile and ceRNA network in SARA cattle, indicating circRNAs’ role in inflammation regulation, thus enhancing our understanding of SARA’s systems biology and potential treatment strategies.

## Simple Summary

High-grain diets can make cows sick with ruminal acidosis, a digestive problem that reduces their health and productivity. Our study aimed to discover how special molecules, called circular RNAs, might affect this illness. In six Holstein cows, we found 65 distinct circular RNAs that change between healthy and sick animals, along with a web of connections to other body molecules. This shows these molecules likely influence ruminal acidosis. Such findings could guide farmers to prevent this disorder, improving cow well-being and boosting dairy and beef benefits for society.

## 1. Introduction

Modern livestock management relies heavily on high-grain diets to enhance productivity and cost-effectiveness in ruminants. However, this practice disrupts rumen microbial balance, as amylolytic bacteria like *Streptococcus bovis* and *Lactobacilli* spp. ferment starches into organic acids, such as lactate, which can accumulate if not efficiently converted to acetate, propionate, and butyrate by lactate-utilizing bacteria like *Megasphaera elsdenii* [1,2]. When fermentation becomes asynchronous, rumen pH drops, initiating ruminal acidosis, a prevalent metabolic disorder in cattle classified into acute (ARA, pH < 5) and subacute (SARA, pH 5.5–5.8) forms [3].

SARA, in particular, affects a significant proportion of cattle, with prevalence estimates ranging from 20% to 40% in beef cattle under intensive feeding systems and similar rates in dairy herds fed high-concentrate diets [4]. This condition, often triggered by small-particle, low-fiber diets, leads to reduced feed intake, diminished milk yield, and compromised animal performance, yet its subtle symptoms make diagnosis challenging [5]. Beyond the rumen, acid absorption disrupts blood pH and increases osmotic pressure, damaging the epithelium, while bacterial lysis releases lipopolysaccharides (LPS), triggering systemic inflammation and dysbiosis [6,7]. Recent efforts to improve ruminal balance have tested oral administration of substances like zeolite, often evaluating outcomes indirectly through metabolism or reproduction [8]. Understanding the mechanisms behind these imbalances could directly assess and evaluate such interventions.

Despite insights into its physiological impacts, the molecular mechanisms driving ruminal acidosis, particularly SARA, remain elusive [9]. Emerging evidence highlights noncoding RNAs, such as circular RNAs (circRNAs), as key regulators of gene expression in metabolic and inflammatory processes—conditions central to SARA—yet their role in this disorder is uncharted [10,11]. Formed by back-splicing into stable, closed-loop structures, circRNAs can sponge miRNAs or interact with proteins, suggesting they may modulate the inflammatory and metabolic shifts observed in affected cattle. The aim of this study was to profile circRNA expression in SARA-affected Holstein cattle and identify their regulatory roles through a ceRNA network, providing insights to mitigate this widespread disorder.

## 2. Materials and Methods

### 2.1. Overview of Study Workflow

**Figure 1:**
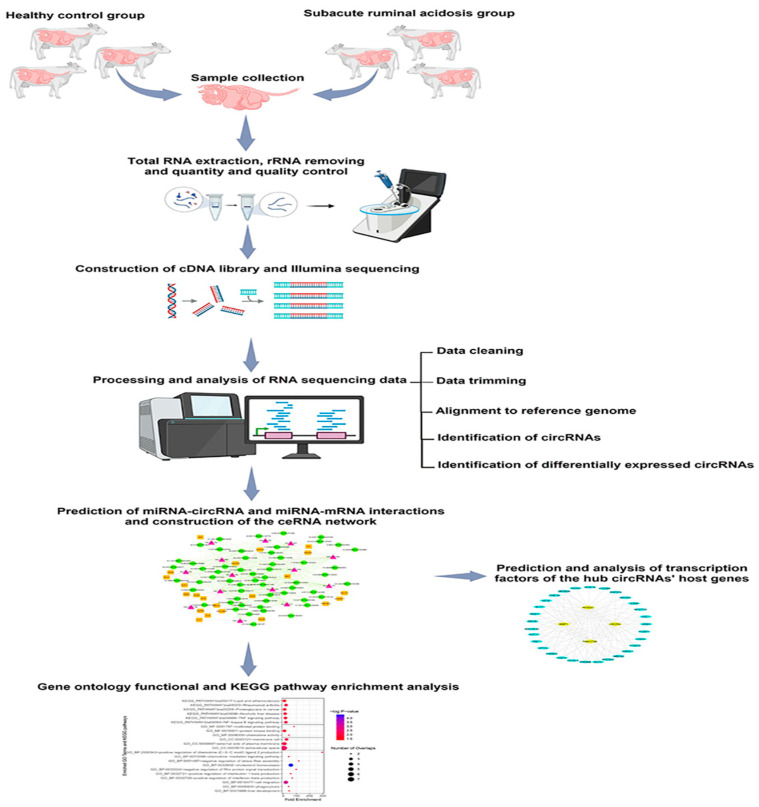
Overview of study workflow.

The workflow of the current study, depicted in Figure 1, involved profiling circRNA expression in subacute ruminal acidosis (SARA) in Holstein cattle. Six animals (three healthy, three SARA-induced) were subjected to a high-concentrate diet, followed by rumen fluid collection, RNA extraction, sequencing, and bioinformatics analysis to identify differentially expressed circRNAs and construct a ceRNA network.

### 2.2. Animals

Samples were collected from six Holstein steers, which were randomly divided into two groups, comprising three experimental and three control animals. The selected steers were 8 months old, with an initial body weight of 250 ± 40 kg. To enhance the steers’ immunity during this period, all animals received a subcutaneous injection of E-selenium.

### 2.3. Induction of Rumen Acidosis and Sample Collection

A specialized dietary regimen was meticulously designed [12] and administered over a predetermined period to induce ruminal acidosis in the experimental group. The ingredients of the diet are detailed in Table 1. Initially, the cattle were allowed ad libitum intake for three days. On the fourth day, their intake was reduced by 50%, and on the fifth day, they were given ad libitum access to either the basal diet or the basal diet supplemented with a wheat/barley pellet, constituting 10% of their previous dry matter intake. This feeding protocol was followed for a total of 130 days. Throughout the research, ruminal pH was continuously monitored to assess and confirm the onset of ruminal acidosis. To confirm the induction of SARA, ruminal pH was measured on Days 1, 30, and 129 using a pH probe inserted through a cannula. Additionally, the concentrations of volatile fatty acids (VFAs) were also measured.

After a 130-day feeding period, tissue samples from the rumen were collected post-slaughter from both SARA-affected and healthy cattle. The samples were taken from the central region of the ventral sac. The samples were rinsed with sterilized PBS buffer and placed into 50 mL tubes containing RNA Later solution (Invitrogen, Carlsbad, CA, USA), then stored at −80 °C until further processing.

### 2.4. RNA Extraction

Total RNA was extracted from the collected samples using Trizol reagent (Invitrogen, Carlsbad, CA, USA), based on the manufacturer’s protocol. About five μg of each extracted total RNA formed the collected samples. We assessed the quality of the extracted total RNA through a two-step process. Firstly, we examined the purity and lack of contamination of the RNA by NanoPhotometer^®^ spectrophotometer (Implen, Munich, Germany) based on in-depth analysis of the RNA’s optical density. Secondly, the total RNA integrity was evaluated by employing the highly sensitive RNA Nano 6000 Assay Kit and the Bioanalyzer 2100 system (Agilent Technologies, Santa Clara, CA, USA). This state-of-the-art assay provided a comprehensive analysis of RNA integrity, ensuring the extracted RNA was intact and free from degradation. Total RNA samples were securely preserved at −80 °C and used for subsequent analyses.

### 2.5. cDNA Library Construction and Sequencing

The extracted total RNA samples meeting the quality criteria were employed to construct cDNA libraries for circRNA. In the first step, we removed the ribosomal RNA by using the Epicentre Ribo-Zero^TM^ rRNA Removal Kit (Thermo Fisher, Waltham, MA, USA). Then, the obtained linear RNA molecules underwent digestion with three units of RNase R per microgram of RNA Kit (Thermo Fisher, Waltham, MA, USA). To provide templates for cDNA synthesis, the resulting RNA fragments were subjected to the addition of A-tails and ligation for sequencing junctions. Construction of the cDNA library was completed by PCR amplification of the modified fragments. During this library construction process, fragments with sizes in the range of 250–300 bp were inserted. Accurate quantification of the effective concentration of the library (≥3 ng/μL) was performed, and then double-end sequencing was performed on the confirmed libraries using the Illumina platform (Illumina, San Diego, CA, USA).

### 2.6. Data Preprocessing for Quality Enhancement in NGS Analysis

To ensure the reliability and accuracy of downstream analyses, a comprehensive data preprocessing pipeline was implemented. Initially, raw sequencing data were subjected to quality assessment using FastQC (version 0.11.7) with default parameters. This step facilitated the identification of low-quality reads and undesirable sequences, such as adapter contaminants, which could compromise subsequent analyses. Following quality assessment, Trimmomatic (version 0.40) [13] was employed to perform rigorous data trimming. This step was critical to mitigate the effects of sequencing errors and biases. Specifically, low-quality reads were removed, adapter sequences were trimmed, and poor-quality bases (e.g., those with Phred scores below a defined threshold) were eliminated.

### 2.7. Alignment to Reference Genome

We aligned the clean reads into the reference genome of *Bos taurus* (BosTau9, available online: https://hgdownload.soe.ucsc.edu/goldenPath/bosTau9/, accessed on 10 November 2018) using HISAT2 software (version 2.0.4). This step aimed to map the reads to their respective genomic locations [14].

### 2.8. CircRNA Identification

A comprehensive approach was employed to minimize false positives during circRNA identification. In this approach, CIRI2 Software (version 1.1.0) [15] was applied with default parameters to screen for circRNAs. CIRI2 has been optimized for crucial steps in circRNA detection. It can accurately deduce the original region for sequencing read segments and effectively distinguish between back-spliced junction (BSJ) reads and forward-spliced junction (FSJ) reads by employing an adapted Maximum Likelihood Estimation (MLE) approach based on multiple-seed matching [15].

Additionally, for accurate quantification of circRNAs, we utilized CIRIquant (v1.1.3), an efficient tool that generates pseudo-reference sequences for identified circRNA transcripts. This tool can seamlessly integrate popular circRNA identification pipelines such as CIRI, CIRCexplorer, and find_circ to provide robust identification of BSJ and FSJ reads and the quantification of circRNAs. It utilizes both the alignment read results to the *Bos taurus* reference genome and pseudo circRNA transcripts, allowing credible quantification of junction ratios for circRNAs and providing an appropriate function for one-stop differential expression analysis of circRNAs [16].

### 2.9. Normalization and Identification of Differential Expression (DE) cicRNAs

To evaluate circRNA expression in the samples, transcripts per kilobase per million mapped reads (TPMs) were employed for normalization. Significant DE-circRNAs were determined using the |log2 Fold Change| ≥ 2 and *p*-value < 0.05 criteria. The EdgeR and DESeq R packages were implemented to analyze the DE-circRNAs between two groups.

### 2.10. Construction of ceRNA Network of circRNA–miRNA–mRNA Interactions

CircRNAs play pivotal roles in biological processes. They can bind to miRNAs or proteins to regulate gene expression and protein function. Furthermore, research has disclosed that circRNAs having ribosome entry sites and open reading frames can undergo translation [17]. Hereupon, to further investigate circRNA functions, it is essential to analyze their miRNA binding sites and coding potential. We investigated miRNA binding sites in circRNA exons by miRanda-3.3a with specific criteria (-sc 140 -en -10; -scale 4 -strict) and determined putative miRNA targets for the circRNAs.

We had previously identified DE-mRNAs in cattle rumen acidosis [18]. We evaluated the potential miRNAs targeting DE-mRNAs by miRTarBase (available online: https://awi.cuhk.edu.cn/~miRTarBase/miRTarBase_2025/php/index.php, accessed on 6 February 2025), an experimentally validated miRNA–target interaction database. Eventually, the RNAs with a complete circRNA–miRNA–mRNA axis were used for circRNA-related ceRNA network construction and analysis by using Cytoscape software (version 3.9.0).

### 2.11. Transcription Factor Analysis for the Host Genes of Hub circRNAs

Transcription factors (TFs) are essential RNA transcription regulators that can bind to specific DNA sequences, often in the promoter DNA sequences. CircRNAs can be regulated by upstream TFs [19]. On the other hand, they can affect the epigenetic state of the promoter regions of their host genes [17]. Accordingly, the promoter sequences of the host genes of the prominent hub circRNAs were collected from the UCSC Genome Browser database (available online: https://genome.ucsc.edu, accessed on 6 February 2025), and then the putative TFs were identified by the AnimalTFDB v4.0 database (available online: http://bioinfo.life.hust.edu.cn/AnimalTFDB, accessed on 6 February 2025). Then, the putative TFs were shared with the DE-mRNAs to find the differentially expressed TFs. The regulatory network of TFs and the host genes was generated using Cytoscape software.

### 2.12. Functional and Pathway Enrichment Analyses

Studies have denoted a strict relationship between circRNAs and their paternal genes, and their function may be associated with their parental linear transcripts [17,20]. Accordingly, investigation of Gene Ontology (GO) terms and Kyoto Encyclopedia of Genes and Genomes (KEGG) pathways may partly explain some function of the circRNAs [19].

Hence, we discovered the genes corresponding to the parental mRNA of each DE-circRNA, and then enriched GO items in the biological process (GO-BP), cellular component (GO-CC), and molecular function (GO-MF) and KEGG pathways by the Database for Annotation, Visualization and Integrated Discovery (DAVID; available online: http://david.ncifcrf.gov, accessed on 6 February 2025) webtool. We also utilized the DAVID online tool to explore the possible roles of the DE-mRNAs from the created ceRNA network via GO and KEGG pathway enrichment. The GO and KEGG items with *p*-value < 0.05 were selected as significant terms. Next, the ggplot2 version 3.5.2 and DOSE R version 3.21 packages were implemented to draw the bubble diagram of the significant GO terms and KEGG pathways.

## 3. Results

### 3.1. Animal Health Monitoring

During the 130-day study, the SARA group exhibited no significant external clinical signs of morbidity, such as lameness or severe diarrhea, consistent with the metabolic nature of subacute ruminal acidosis (SARA). Their demeanor remained normal throughout the study. As the cattle were part of a feedlot (fattening) program, they were sent to the slaughterhouse at the end of the study, following standard industry practices.

### 3.2. Rumen Metabolic Parameters and circRNA Quality Control

The study showed significant changes in rumen parameters due to SARA diets (Table 2). SARA cows had consistently lower pH (5.18–5.61 vs. 5.81–5.83 in controls, *p* < 0.05) and higher propionate (18.11 vs. 14.45 mM, *p* < 0.01) and Valerate (1.45 vs. 1.25 mM, *p* < 0.001). Butyrate was lower in SARA cows (13.22 vs. 14.21 mM, *p* < 0.05), while NH_3_-N was higher (7.71 vs. 6.96 mg/dL, *p* < 0.05). These changes confirm SARA’s impact on rumen fermentation and nitrogen metabolism.

**Figure 2:**
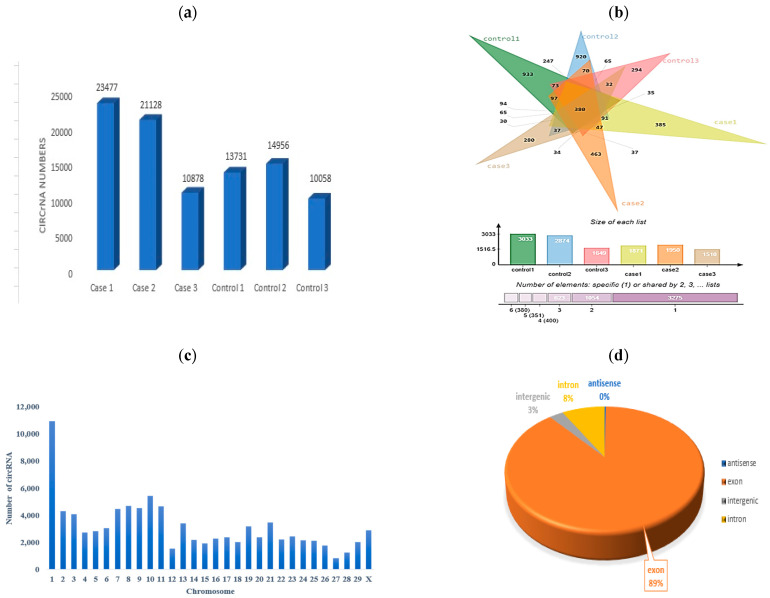
Statistics of identified circRNAs. (**a**) Number of the circRNAs in SARA and control samples. (**b**) Venn diagram and bar chart of the numbers of unique and shared circRNAs of each sample. (**c**) Number of circRNAs on each chromosome. (**d**) Type distribution.

In the present study, we implemented CIRIquant for precise quantification of circRNAs in the RNA-Seq data to reduce the likelihood of false positives associated with BSJ reads. We obtained 426,352,948 raw reads from our RNA-seq data. After preprocessing, including removing low-quality reads, poly-N sequences, and reads with adapters, 188,456 clean reads were retained. Next, the clean reads were aligned into the Bos taurus reference genome, and about 95% of them were successfully mapped. Each sample yields approximately 31,409 raw reads for the dataset (Table 3). Read counts are provided for each sample, with ”2×” signifying case sample data duplicated for comparison (Table 3 and Figure 2).

The origin of circRNAs in the six samples indicated that circRNAs were from exonic, intergenic, and intronic regions, with the highest number of circRNAs placed in exonic regions, followed by intronic and intergenic regions. This observation underscores that exon splicing is the primary mechanism behind circRNA generation, which is consistent with the established understanding of circRNA biogenesis.

### 3.3. Identification of DE-circRNAs in SARA-Affected Cattle

**Figure 3:**
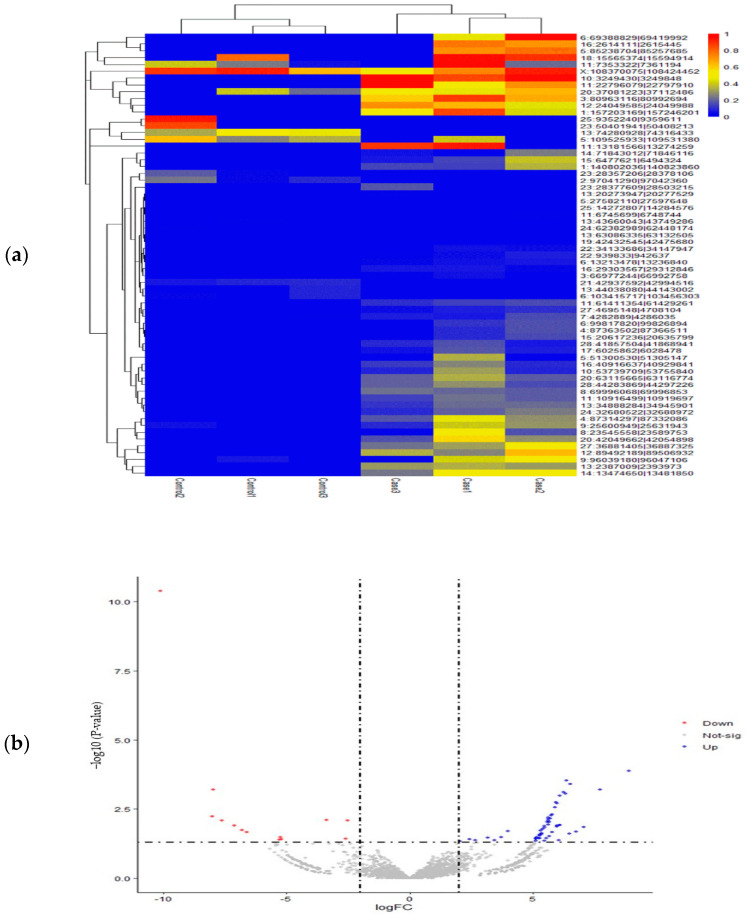
Expression data analysis of Differential Expression of Circular RNAs (DE-circRNAs). (**a**) Heatmap illustrating the distribution of differentially expressed circRNAs (DE-circRNAs) in different groups and samples, highlighting the expression patterns and relative abundance of these molecules. (**b**) Volcano plot displaying DE-circRNAs. That blue points represent significantly upregulated circRNAs, red points show downregulated circRNAs, and gray points indicate non-significant circRNAs. Dashed lines mark significance thresholds.

Using fold change and an adjusted significance threshold, we determined differentially expressed circRNAs (DE-circRNAs) in cattle affected by SARA. The EdgeR analysis revealed 65 circRNAs with distinct expression patterns across the samples. Among these, 52 circRNAs were significantly upregulated, showing a marked increase in their expression levels (Figure 3). Table 4 presents the top 20 upregulated circRNAs, which are the most notable candidates in SARA-affected cattle. It also includes downregulated circRNAs that show decreased expression levels compared to the controls.

### 3.4. Analysis of circRNA–miRNA–mRNA Network

**Figure 4:**
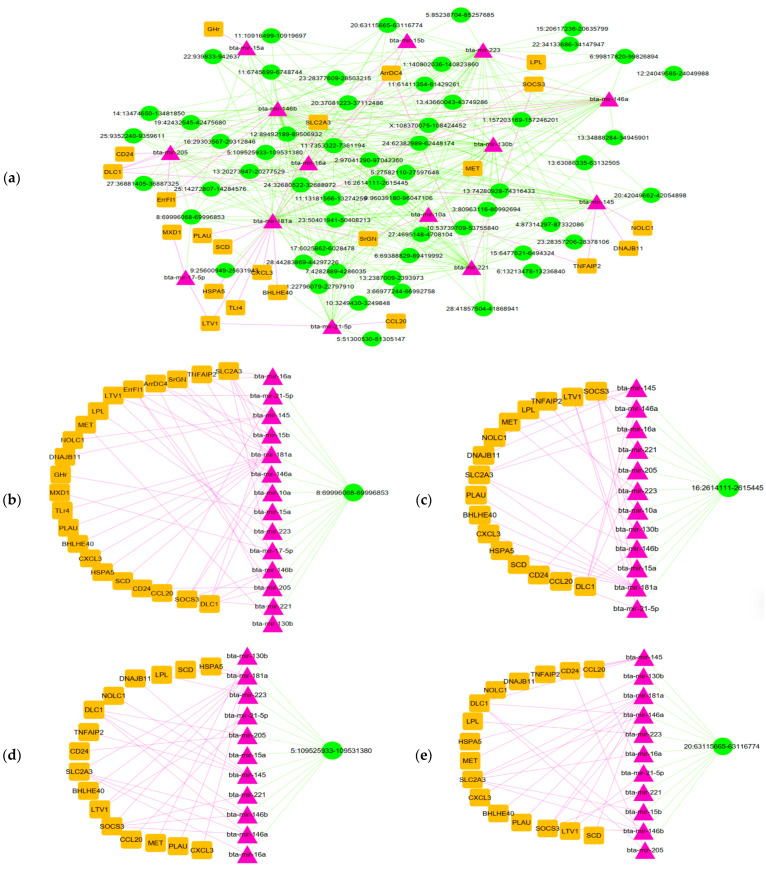
The ceRNA network of circRNA–miRNA–mRNA interactions and its top four subnetworks. (**a**) CircRNA–miRNA–mRNA network. (**b**) Subnetwork from circRNA 8:69996068-69996853. (**c**) Subnetwork from circRNA 16:2614111-2615445. (**d**) Subnetwork from circRNA 5:109525933-109531380. (**e**) Subnetwork from circRNA 20:63115665-63116774.

CircRNAs serve as main regulators in the competing endogenous RNA (ceRNA) network, influencing gene expression by acting as miRNA sponges. Hence, we employed the miRanda software (v3.3a) to identify interactions between differentially expressed (DE) circRNAs and miRNAs. Furthermore, validated miRNA–DE-mRNA interactions from our prior research were examined using the miRTarBase database. The circRNA–miRNA and mRNA–miRNA networks were integrated by Cytoscape, ensuring that incomplete circRNA–miRNA–mRNA axes were excluded from the final analysis. This process generated a ceRNA network including 57 circRNAs, 14 miRNAs, and 22 mRNAs (Figure 4a). Based on degree scores, the leading hub miRNAs were bta-miR-146b, bta-miR-181a, bta-miR-223, and bta-miR-130b, while the prominent hub mRNAs included SLC2A3, SOCS3, DLC1, and ARRDC4. The top four circRNA–miRNA–mRNA subnetworks are visualized in Figure 4b.

### 3.5. Analysis of TFs Associated with Host Genes of Hub circRNAs

**Figure 5:**
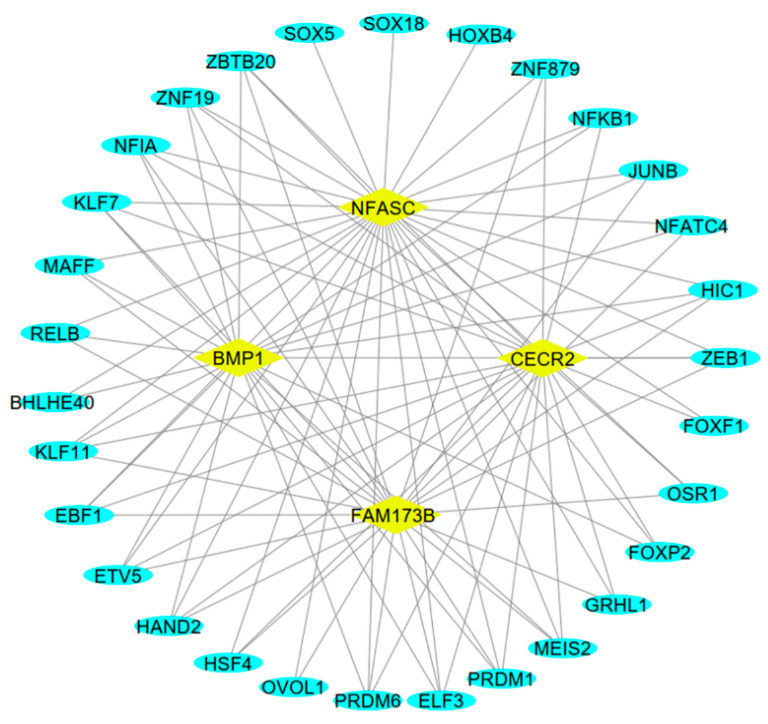
The regulatory network of the parental genes and transcription factors of the hub DE-circRNAs. The predicted transcription factors with differential expression are presented by oval blue nodes, and the host genes are indicated by rhombus yellow.

Since the regulation of circRNA expression is regulated by upstream TFs, we evaluated the sequences of promoter regions of the host genes of circRNA 8:69996068-69996853 (BMP1), circRNA 16:2614111-2615445 (NFACS), circRNA 5:109525933-109531380 (CECR2), and circRNA 20:63115665-63116774 (FAM173B) with the AnimalTFDB v4.0 database for TF prediction and determined the common TFs between predicted TFs and DE-mRNAs. Eventually, we identified 30 differentially expressed TFs associated with the host genes of the hub circRNAs. The regulatory network of the host genes and differentially expressed TFs consisted of 34 nodes and 95 edges (Figure 5). Notably, the network analysis exhibited that ZEB1, KLF11, NFIA, KLF7, HIC1, MEIS2, ELF3, ZBTB20, ETV5, HAND2, PRDM6, EBF1, ZNF19, and PRDM1 TFs interacted with all four host genes.

### 3.6. Functional and Pathway Enrichment Analyses

Considering the strong association between circRNA functions and their parental genes, we conducted enrichment analyses, consisting of Gene Ontology (GO) terms and KEGG pathways, on the genes corresponding to the parental mRNAs of the DE-circRNAs to explore the roles of circRNAs in cattle affected by SARA. GO enrichment analysis using the DAVID tool revealed that the host genes of the DE-circRNAs were mainly associated with the biological process (GO-BP) of “actin polymerization or depolymerization”, the cellular component (GO-CC) of “focal adhesion”, and the molecular function (GO-MF) of “ketosteroid monooxygenase activity”. In terms of KEGG pathway enrichment, “regulation of actin cytoskeleton” was identified as the main enriched pathway.

**Figure 6:**
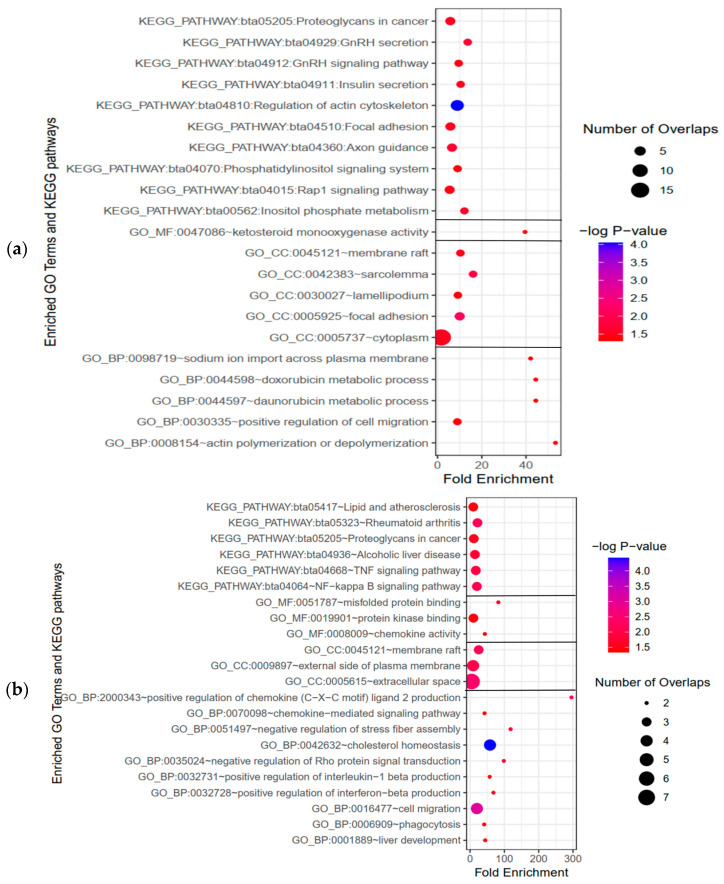
Enrichment analyses of GO terms and KEGG pathways. (**a**) GO term and KEGG pathway enrichment of parental genes of identified DE-circRNAs, covering molecular functions (GO_MF), cellular components (GO_CC), and biological processes (GO_BP). (**b**) GO term.

Additionally, we analyzed the functional roles of mRNAs from the constructed ceRNA network using GO and KEGG pathway enrichment. The results highlighted significant enrichment in GO-BP for “cholesterol homeostasis”, GO-CC for “membrane raft”, GO-MF for “protein tyrosine kinase activator activity”, and the KEGG pathway for “NF-kappa B signaling pathway”. The bubble charts depicting the significant GO terms and KEGG pathways are represented in Figure 6.

## 4. Discussion

We analyzed the obtained circRNA expression data and identified a total of 65 DE-circRNAs in cattle rumen tissues, of which 52 of them showed significant upregulation and 13 of them showed significant downregulation.

Since the circRNA functions may be associated with their parental genes, we employed functional enrichment analyses on the DE-circRNA host genes. These enrichment analyses implied that the parental genes were significantly enriched in GO-CC terms of “focal adhesion”, “cytoplasm”, “lamellipodium”, “sarcolemma” and “membrane raft”, GO-BP of “actin polymerization or depolymerization”, and “positive regulation of cell migration”, as well as KEGG pathways of “Rap1 signaling pathway”, “regulation of actin cytoskeleton”, and “focal adhesion”.

Our findings strongly align with the results of a study by Wang et al. [21], which evaluated the responses of yak rumen epithelial cells to conditions mimicking SARA etiology, such as high concentration of short-chain fatty acids (SCFAs), carboxylic acids produced through the fermentation of undigested polysaccharide by gut bacteria. Their study demonstrated that SCFA accumulation disrupts actin cytoskeleton organization and focal adhesion integrity, leading to compromised epithelial barrier function [21]. Thus, the DE-circRNAs may contribute to regulation of cell adhesion, junction stability, and cell migration, leading to ruminal epithelial barrier dysfunction. Notably, Wang et al. [21] also reported upregulated purine and amino acid metabolism in response to SCFA, suggesting that these molecular changes may initially serve as adaptive mechanisms to maintain cellular homeostasis under acute stress. Similarly, low pH-induced activation of complement and coagulation cascades could reflect a protective response to mitigate tissue damage.

Moreover, the DE-circRNA host genes were significantly enriched in “sodium ion import across plasma membrane” GO-BP term. It is well established that sodium is transported across the rumen epithelium in rumen acidosis, leading to elevated blood sodium levels. This process is primarily mediated by sodium-hydrogen exchangers (NHEs), particularly NHE3, which is known to be downregulated under acidic conditions, further disrupting ionic homeostasis in the rumen epithelium [22,23]. Additionally, the enrichment analysis of the circRNA parental genes highlighted “inositol phosphate metabolism” and “phosphatidylinositol signaling system” KEGG pathways. These pathways are involved in intracellular signaling, mediating responses to extracellular stimuli through rapid turnover, thereby regulating various cellular processes, such as apoptosis, proliferation, and cytoskeletal rearrangement [24]. Hence, the role of DE-circRNAs should be further examined within the framework of these metabolic and signaling pathways to better understand their implications in ruminal acidosis.

Moreover, the identified KEGG pathways of “insulin secretion”, “GnRH signaling pathway”, and “GnRH secretion” suggest potential endocrine disruptions in response to SARA. It has been demonstrated that SARA in ruminants can lead to insulin resistance, resulting in hyperinsulinemia and elevated blood glucose levels [25]. Additionally, multiple studies have reported that changes in extracellular pH are often accompanied by intracellular pH alterations, which can influence insulin secretion and contribute to insulin resistance [26]. These metabolic alterations may further exacerbate inflammatory responses and compromise reproductive efficiency in cattle. Given that insulin stimulates gonadotropin-releasing hormone (GnRH) secretion [27], it is plausible that dysregulated circRNA expression could impact reproductive pathways via insulin–GnRH interactions. Since GnRH is important in the regulation of mammalian reproductive cycles, circRNA dysregulation in this context may hold critical implications for fertility in dairy and beef cattle [28].

To further elucidate the regulatory landscape of these circRNAs, we constructed a circRNA-mediated ceRNA network and identified the top four circRNA–miRNA–mRNA subnetworks. Key circRNAs such as circRNA 8:69996068-69996853, circRNA 16:2614111-2615445, and circRNA 5:109525933-109531380 emerged as hub regulators within this network, interacting with crucial miRNAs like bta-miR-181a, bta-miR-146a, bta-miR-130b, and bta-miR-223. These interactions suggest a complex regulatory mechanism, particularly in immune responses and metabolic adaptations to SARA. Notably, our findings are in concordance with a study by Pacifico et al., who reported downregulation of miR-146a in the rumen epithelium of Holstein cows fed a high-grain diet [29]. This aligns with our ceRNA network results, where 63 circRNAs interacted with miR-146a, influencing key immune-related targets such as SOCS3, SLC2A3, ERRFI1, and DLC1. Furthermore, 43 circRNAs interacted with miR-181a, regulating immune and inflammatory mediators such as TLR4, SRGN, SOCS3, and CXCL3 [30,31,32].

Additionally, circRNA-associated ceRNA networks revealed a potential role of lipid metabolism and inflammatory control. Specifically, 39 circRNAs were shown to interact with miR-223, targeting LPL mRNA, while 38 circRNAs interacted with bta-miR-130b, targeting MET mRNA [33]. Given that miR-223 suppresses lipid accumulation and inflammatory cascades through NF-κB inhibition, its modulation by circRNAs suggests a novel axis of metabolic–inflammation interplay in SARA [34,35].

Since circRNA expression may also be regulated by upstream transcription factors (TFs), we analyzed the promoter sequences of the host genes of circRNA 8:69996068-69996853 (BMP1), circRNA 16:2614111-2615445 (NFACS), circRNA 5:109525933-109531380 (CECR2), and circRNA 20:63115665-63116774 (FAM173B). Our analysis identified 30 differentially expressed TFs, among which ZEB1, KLF11, NFIA, KLF7, and PRDM1 were highly correlated with all four hub circRNAs, reinforcing their potential role in inflammation and epithelial function [36,37]. Given that NF-κB transcription factors regulate inflammatory responses, further research is warranted to assess their direct involvement in circRNA-mediated immune modulation [38,39].

Finally, functional enrichment analyses of mRNAs from the ceRNA network reinforced the inflammatory nature of SARA pathology. The most significantly enriched pathways included “NF-kappa B signaling pathway” and “TNF signaling pathway”, both of which are crucial mediators of immune activation and cytokine production [40,41]. In agreement with Zhao et al.’s findings, we observed that these pathways contribute to an increased expression of pro-inflammatory cytokines such as IL-1β, IL-6, and TNF-α in SARA-affected ruminal tissue [42].

Thus, future investigations should focus on validating these regulatory mechanisms, particularly the circRNA-mediated modulation of NF-κB and TNF signaling in SARA progression. A key limitation of this study is its small sample size of six animals, with three per group (healthy and SARA), which may not fully reflect the biological variability in cattle due to genetic, environmental, and pathophysiological factors. This exploratory study, limited by resources, applied strict criteria (log2 Fold Change ≥ 2, *p*-value < 0.05) to identify 65 differentially expressed circRNAs (DE-circRNAs), yet the modest number may constrain statistical power and risk bias. Nevertheless, these novel findings on circRNA expression and ceRNA networks in SARA provide a valuable starting basis for future studies, ideally with 6–8 animals per group to enhance robustness. To our knowledge, the differentially expressed circRNAs, circRNA-mediated ceRNA network, and their role in cattle rumen acidosis derived from this study have not been previously published. Therefore, in this paper, we have demonstrated the circRNA expression profile of samples from cattle with SARA and healthy controls and identified 65 DE-circRNAs. Additionally, we present a circRNA–miRNA–mRNA ceRNA network using the obtained circRNAs, differentially expressed mRNAs from our previous study, and the predicted targets of miRNAs from miRanda and miRTarBase. The constructed ceRNA network consists of 57 circRNAs, 14 miRNAs, and 22 mRNAs. We predicted the functions of the circRNAs by using GO term and pathway enrichment of their parental genes and the mRNAs from the circRNA-mediated ceRNA network. Additionally, we identified 30 differentially expressed TFs associated with the parental genes of the hub circRNAs.

## 5. Conclusions

Our findings underscore the pivotal role of circRNAs in the inflammatory and metabolic disruptions observed in SARA. By modulating key immune, metabolic, and epithelial barrier pathways, circRNAs may serve as novel biomarkers or therapeutic targets for mitigating SARA-related disorders in cattle. However, it is important to note that this study is preliminary, based on a small sample size. Further research involving a larger number of animals and additional molecular parameters related to the development of SARA and circRNA adaptation is necessary. Understanding these mechanisms can offer valuable insights into potential therapeutic strategies to alleviate inflammation and its consequences in cattle experiencing rumen acidosis.

## Figures and Tables

**Table 1:** The ingredients in the diet’s composition used in this study.

Ingredients	Control Diet (%)	High-Concentrate Diet (%)
Alfalfa hay	10.5	10.5
Corn silage	14.5	14.5
Barley grain	34	34
Corn grain ground	5.3	23.3
Soybean meal	9	9
Wheat bran	10.5	3
Rice bran	6	2.5
Sucrose	5	-
Fat powder	2.5	-
Supplement	3.2	3.2

**Table 2:** Rumen pH, volatile fatty acids (VFAs), and ammonia concentrations in control versus subacute ruminal acidosis (SARA) diets.

Item	Case (Mean ± SEM)	Control (Mean ± SEM)	*p*-Value
pH_1	5.61 ± 0.21	5.83 ± 0.06	0.042 *
pH_30	5.56 ± 0.23	5.80 ± 0.12	0.038 *
pH_129	5.18 ± 0.04	5.81 ± 0.28	0.009 **
Acetate (mM)	69.70 ± 0.82	71.74 ± 0.78	0.098 ^ns^
Propionate (mM)	18.11 ± 0.49	14.45 ± 0.65	0.003 **
Butyrate (mM)	13.22 ± 0.17	14.21 ± 0.11	0.012 *
Valerate (mM)	1.45 ± 0.07	1.25 ± 0.09	0.001 ***
NH₃-N (mg/dL)	7.71 ± 0.21	6.96 ± 0.08	0.021 *

* *p* < 0.05, ** *p* < 0.01, *** *p* < 0.001, ns = not significant (*p* ≥ 0.05).

**Table 3:** Quantitative results of circRNAs obtained using CIRIquant in case (SARA cattle) and control (Healthy Cows) Samples.

Sample	Case 1	Case 2	Case 3	Control 1	Control 2	Control 3
Count	23,477	21,128	10,878	13,731	14,956	10,058
Total (2×)	46,954	42,256	21,756	27,462	29,912	20,116

**Table 4:** Differentially expressed circRNAs in case and control group.

CircRNA Id	logFC	*p*-Value	Up/Down Change
11:13181566|13274259	8.922037	0.000134	up
28:41857504|41868941	6.357273	0.000294	up
13:2387009|2393973	6.531094	0.000392	up
25:14272807|14284576	7.736988	0.000626	up
3:80963116|80992694	6.255852	0.000763	up
13:34888284|34945901	6.334839	0.000889	up
22:939833|942637	6.121243	0.001056	up
12:24049585|24049988	5.952984	0.001773	up
4:87314297|87332086	5.99522	0.002001	up
10:3249430|3249848	5.898283	0.002758	up
20:63115665|63116774	5.785773	0.004924	up
17:6025862|6028478	5.760129	0.005367	up
6:13213478|13236840	5.648043	0.006443	up
13:20273947|20277529	5.724471	0.006985	up
16:40916637|40929841	5.652667	0.007216	up
8:69996068|69996853	5.626879	0.008086	up
27:36881405|36887325	5.678958	0.009176	up
11:61411354|61429261	5.589611	0.009462	up
11:22796079|22797910	5.653773	0.011334	up
14:71843012|71846116	6.099256	0.012034	up
13:74280928|74316433	−10.1139	4.14 × 10^−11^	Down
23:28357206|28378106	−7.97577	0.000617	Down
24:62382989|62448174	−8.02504	0.005943	Down
5:109525933|109531380	−3.39204	0.00778	Down
X:108370075|108424452	−2.5376	0.008075	Down
25:9352240|9359611	−7.61768	0.008212	Down
23:50401941|50408213	−7.10743	0.0126	Down
13:44038080|44143002	−6.81868	0.018544	Down
13:43660043|43749286	−6.61989	0.021539	Down
21:42937592|42994516	−5.2342	0.033789	Down
11:7353322|7361194	−2.5856	0.037708	Down
2:97041290|97042360	−5.21853	0.038482	Down
6:103415717|103456303	−5.29858	0.0415	Down

## Data Availability

The data that support the findings of this study are available from the corresponding author upon reasonable request.
